# Integrating Spatial Working Memory and Remote Memory: Interactions between the Medial Prefrontal Cortex and Hippocampus

**DOI:** 10.3390/brainsci7040043

**Published:** 2017-04-18

**Authors:** Ryan A. Wirt, James M. Hyman

**Affiliations:** Department of Psychology, University of Nevada Las Vegas, 4505 S. Maryland Pkwy, Las Vegas, NV 89154, USA; ryan.wirt@unlv.edu

**Keywords:** hippocampus, medial prefrontal cortex, spatial working memory, remote memory, Theta

## Abstract

In recent years, two separate research streams have focused on information sharing between the medial prefrontal cortex (mPFC) and hippocampus (HC). Research into spatial working memory has shown that successful execution of many types of behaviors requires synchronous activity in the theta range between the mPFC and HC, whereas studies of memory consolidation have shown that shifts in area dependency may be temporally modulated. While the nature of information that is being communicated is still unclear, spatial working memory and remote memory recall is reliant on interactions between these two areas. This review will present recent evidence that shows that these two processes are not as separate as they first appeared. We will also present a novel conceptualization of the nature of the medial prefrontal representation and how this might help explain this area’s role in spatial working memory and remote memory recall.

## 1. Introduction 

Spatial memory is one of the most well-studied functions in neuroscience. While extensive research has firmly established that the medial temporal lobes, including the hippocampus (HC), are a central node for spatial memory function; recently, there has been a growing line of evidence suggesting that the medial prefrontal cortex (mPFC), including the anterior cingulate cortex (ACC), also plays an integral role. Furthermore, research has shown that it is the interactions between these two areas that may be at the heart of successful encoding and retrieval of spatial information. This idea has arisen from two disparate research streams: one into spatial working memory and the other into the consolidation of long-term spatial memories. Studies of spatial working memory concentrate on ‘online’ processing of trial specific spatial information and examine how information is shared between the two areas. These studies have primarily employed in vivo electrophysiology to show that neurons in the two areas fire synchronously during working memory tasks [1,2,3,4]. Consolidation studies examine the possible transference of spatial information from the medial temporal lobes to the prefrontal cortex. It is argued that, as time passes, memories are transferred, in some fashion, from one area to another, so more recent memories are dependent on the medial temporal lobes and more remote memories on the mPFC [5,6]. This review will examine both spatial working memory and long-term memory interactions between the mPFC and HC, concentrating on whether these processes are inherently separate or merely two sides of the same coin. We will first present evidence of online spatial processing in neuronal ensembles in both areas. Next, we will detail work that reveals that neural oscillations help to coordinate activity in the two areas during spatial working memory tasks. Then, we will examine the evidence from consolidation studies. Finally, we will attempt to link together these two research streams. 

## 2. Processing and Encoding Information about Space and Context

The ability to encode information about our surroundings is crucial for survival; however, this can be a complex cognitive process. Spatial learning requires immediate processing of incoming information that is reliant on an interconnected network of multiple brain areas. Research has repeatedly established that the HC and surrounding medial temporal areas are crucial for processing spatial information (for review, see [7]). A classic example of this effect can be seen when the HC is lesioned and animals are unable to successfully navigate through space [8]. Additionally, a vast wealth of electrophysiological data has shown robust spatially-related responses in hippocampal neurons [9,10], temporal sequence or time-related responses [11], stimulus specific responses [12,13], and contextual responses [14]. While the data implicating the mPFC’s role in spatial processing has not been as abundant, previous work has shown this area to be fundamental for spatial learning [15]. Below, we will highlight recently reported electrophysiological studies that have shown similarities and differences between the HC and mPFC during the active processing of spatial information.

### 2.1. Space: The Hippocampus

**Figure 1:**
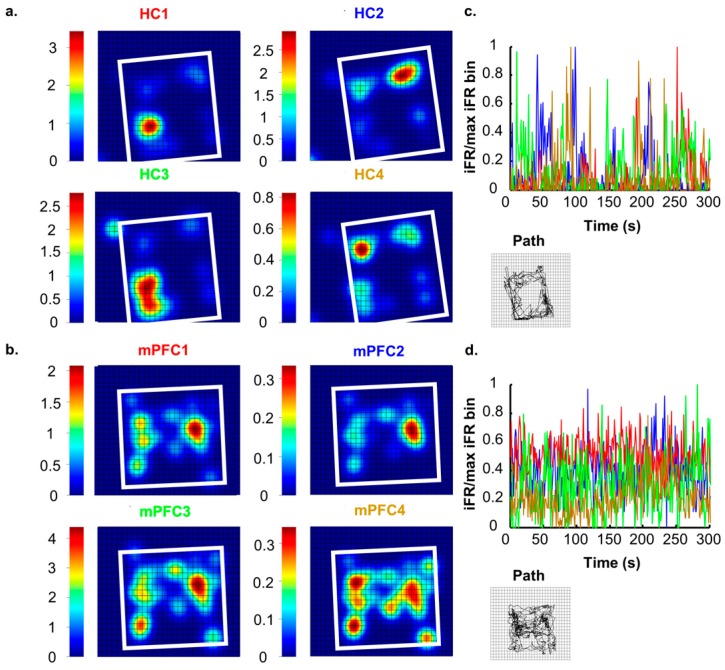
Spatial tuning of hippocampus (HC) & medial prefrontal cortex (mPFC) single-units. (**A**,**B**) heat relief plots of representative example hippocampal (**a**) and mPFC (**b**) unit firing rates during free exploration. Right, spatial position plots showing animal path during recordings. Note that the firing rate by position plots for hippocampal units all show clear well-defined firing fields, or specific locations within the environment where the cells were selectively active. However, mPFC cells were generally more active throughout the entire environment and thus did not have clear place fields. In fact, the few isolated locations in the environment, where mPFC cells were more active, seemed to merely reflect the amount of time spent in those locations more than anything, as one can see in the XY position traces on the right; (**c**,**d**) firing rates of example neurons over time. In each plot, normalized firing rates are shown for the four example neurons from the two areas. Time in seconds in the *x*-axis and proportion of maximum firing is on the *y*-axis. Line color corresponds to the color of the text above the spatial firing rate plots in (**a**,**b**). These plots reveal the inherent firing characteristics of cells in the two areas. The sparse coding scheme found in the HC suggests that a large amount of information is stored by single units, whereas mPFC neurons seem to carry very little environmental information scattered over entire ensembles of neurons. Notice how in (**c**), at most points in time, only a single hippocampal cell is active, as only one colored line at a time rises above 0 and the rest are all at or near 0. In addition, note that when cells become active, they tend to fire close to their maximum firing rate. This type of firing is emblematic of a sparse coding scheme, where only a few neurons are part of the information ensemble and each neuron represents highly specified information. Cells in the mPFC on the other hand react very differently. These cells are maintaining elevated firing rates consistently over the entire window, but these rates are most often only ~50% of the maximum rate. Thus, at most moments, the animal was only in the place field of a single HC cell and, in turn, only that cell was firing, while, in the mPFC, most cells were simultaneously active regardless of the animal’s precise location within the environment. The analyses presented here were performed on data first reported in [19]. Please see the original article for methodological details.

Neurons in the rodent HC are highly attuned to processing and encoding information about the surrounding world. Successful navigation through a spatial task requires the constant updating of currently available contextual cues and access to previously stored information [16]. Neuronal ensembles in the HC are well suited to this, and it has previously been reported that these cells communicate specific contextual information about environments [9,17,18,19,20,21]. Hippocampal ensembles create a unique neural representation of each spatial environment that consists of many individual place fields that together tile the entire environment ([22,23,24]; see Figure 1A,C). When moved to another environment, the activity states across the hippocampal network shift and another neural representation arises that is consistent with the second environment [9,25,26]. In perhaps the most extreme example of dynamic shifts in neuronal environmental representations, Jezek and colleagues [27] recorded neurons from the HC during a task in which subjects were ‘teleported’ between two distinct but familiar environments. Teleportation was accomplished by allowing subjects to forage in two environments that were the same in every way except for lighting patterns (horizontal or vertical). Throughout exposures, researchers identified unique hippocampal place fields for each environment. After the environments had become completely familiar to the subjects, the test phase (teleportation) was initiated. The test phase started like each of the other exposures, with the subject placed in the environment and allowed to explore freely. However, after a set amount of time, the researchers changed the wall lights to the opposing pattern. The change in light pattern caused place fields to flicker (back and forth firing between place fields previously observed in each environment) before settling into a representation that resembled the current environment. These results indicate that neurons in the HC are actively encoding and processing visual information about surroundings, even when this change happens rapidly and unexpectedly. 

Showing that hippocampal networks have unique firing patterns for different environments does provide circumstantial evidence for the existence of a hippocampal spatial engram, and this idea was furthered when experimenters used network activity itself to create new spatial codes in the hippocampus [28]. Animals were exposed to a neutral environment and the engram for that environment was tagged. Then, animals were shocked in a different environment and the neutral environment representation was simultaneously stimulated, thus meshing the representations of the shock and neutral environments. This manipulation leads to increased freezing behavior in the neutral environment, and the authors concluded that they had created a false memory by manipulating the engram of the neutral environment. While there are valid criticisms to the approach employed in this study (effectiveness of doxycycline viral suppression, temporal control of viral expression), this study does reinforce the idea that a hippocampal engram exists and that it is unique for different environments. While these two studies are just the tiniest snippet of the full breadth of hippocampal spatial processing research, we believe that they are exemplars that fully display the HC’s most well-known capability.

### 2.2. Context: The Medial Prefrontal Cortex

Spatial processing is obviously an important part of behavior and the HC clearly plays a fundamental role, but like any neurological process, one brain area is not solely responsible; rather, a widely distributed network of structures is involved. Many other brain areas have been implicated in spatial processing including visual areas [29], parietal areas [30], auditory areas [31], the amygdala [32], the striatum [33], the mPFC [34], and even the cerebellum [35]. When analyzing neural networks involved in cognitive processes, some areas play specific roles and others may serve as more central processing nodes. For example, the amygdala is important for contextual fear learning [36], but not emotionally neutral spatial processing [37]. Furthermore, mPFC has also been implicated in specific spatial tasks, both requiring and not requiring working memory [38,39,40]; acquisition of fear learning [41], and spatial sequence tasks [42], and these tasks are quite disparate and all together encompass the wide breadth of spatial processing. This suggests that the mPFC may play a more central role in spatial information processing than other areas of the spatial network, such as the amygdala. 

**Figure 2:**
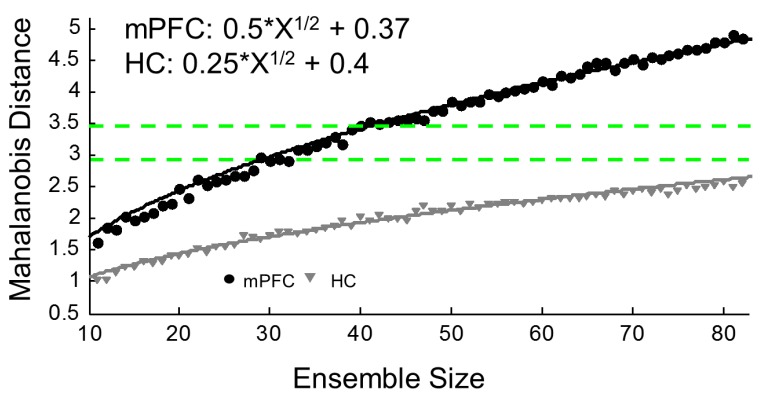
Coding Schemes of the medial prefrontal cortex (mPFC) and hippocampus (HC). Information content by ensemble size. This analysis compares the separation in higher dimensional space between the neural representations of two distinct spatial environments. In these sessions, animals spent time in two empty open field environments and this analysis examines how neuronal ensembles responded to the change in environments. To create this plot, neuronal firing rates from last 200 s in environment A were compared to activity from the first 200 s in environment B from all mPFC and HC sessions, respectively, were combined to create four matrices. Then, *n* neurons (range = 10–83) were randomly selected (without replacement within draws and with replacement between draws) and the Mahalanobis distance between environment periods was calculated using the same randomly drawn neurons for both periods. This process was repeated 100 times for each ensemble size. The *x*-axis shows the size of each ensemble and the *y*-axis is the mean of the Mahalanobis distances between environments for each step. Lines show the fit lines as defined by the inset equations. mPFC values are in black and HC are grey. The green dotted lines show the mean ± standard error of the mean Mahalanobis distance from the original mPFC “no-task” session ensembles. mPFC ensembles contain a much more distributed coding scheme of unique environments. The mPFC fit slope is substantially steeper and even ensembles of over 80 HC neurons have between environment distances less than the original mPFC session mean minus the SEM. The analyses presented here were performed on data first reported in [19]. Please see the original article for methodological details.

Though individual mPFC cells tend to contain very little purely spatially-related information ([43]; see Figure 1B,D), a more complete contextual signal arises when large ensembles of units are recorded, and, in fact, mPFC ensembles contain more robust context information than hippocampal ensembles ([19]; see Figure 2). While our lab has found that mPFC units differentiate whole contexts, they carried very little specific information about locations within environments, making the mPFC spatial signal more a pure context signal. This can be seen in the large changes in mPFC ensemble states that occurred as the animal moved or was placed from one context to another. In fact, this effect was so consistent and powerful that we were able to decode an animal’s environmental context based solely on mPFC ensemble states [19]. If we expand our concept of a context to one that does not necessarily need to be only spatial, we see that, in fact, similar results, i.e., large changes in mPFC ensemble states, have also been found with changes in behavioral sequence [42,44], task phase [45], rules [46], or reward location [47]. All of these differences can be viewed as changes of a larger context representation; in many cases, the context may be cognitively-based, or, in the absence of a clear task, it may be spatially-based. Nonetheless, time after time, and across species, recording studies have shown that within mPFC ensembles a larger signal is represented, some have called this a “task set” [48], while we have referred to it as a context representation [19]. In either case, the importance of this unifying context signal becomes most apparent when we examine how the mPFC affects hippocampal spatial processing below.

## 3. Information Sharing

In 1994, Gray theorized that neural networks involving multiple brain areas could share information by oscillating synchronously [49] (for review, see [16]). In rodents, researchers had already identified theta rhythm (4–8 Hz) as an integral oscillation within the HC that is necessary for accurate learning and recall [4,50,51,52,53]. Theta waves accompany many rodent behaviors including: running [54,55,56], sniffing [55,57], conditioned freezing [58], orienting [59], and exploration [60]. More recently, much research has shown that interactions between other brain areas and the HC occur at theta frequencies, helping to form functional connections [61]. For example, the amygdala [62] and cerebellum [63] are synchronized to hippocampal theta when responding to a conditioned stimulus. Likewise, during spatial working memory tasks, the nucleus accumbens [64] and the mPFC are entrained to hippocampal theta [1,2,3,4]. Complex behaviors are thought to rely upon these types of interactions between brain areas, giving animals the ability to complete difficult tasks [48] and when communication is severed between areas there are profound deficits in behavior and recollection [65,66,67]. This section will attempt to explain the significance of interactions between the mPFC and HC by highlighting recent reports that have utilized multiple behavioral paradigms relating to working memory and the active processing of spatial information. 

### 3.1. Functinal Anatomy HC-mPFC

**Figure 3:**
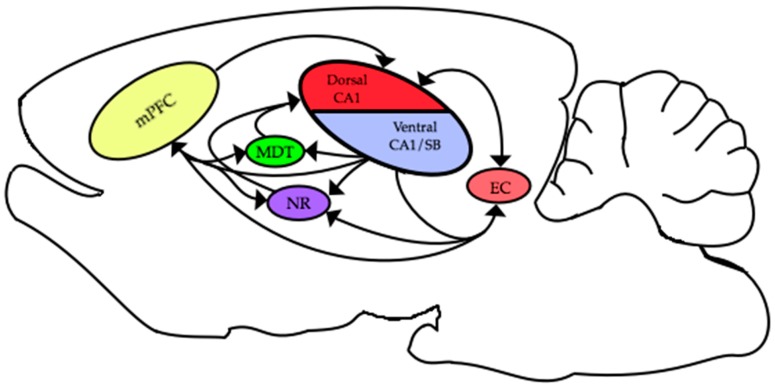
Relevant connections between the hippocampus (HC) and medial prefrontal cortex (mPFC). medial prefrontal cortex = mPFC; nucleus reuniens = NR; mediodorsal thalamic nuclei = MDT; subiculum = SB; CA1 = dorsal (top) ventral (bottom).

The functional connections between different areas are thought to be critical for many cognitive and behavioral processes. The HC and mPFC are connected by multiple pathways that enable these two areas to interact and engender successful behavioral and cognitive performance. In rodents, there are excitatory monosynaptic projections originating in ventral CA1/subiculum to prefrontal areas [68,69,70]. Additional, hippocampal efferent pathways from these same areas project to the entorhinal cortex [71], nucleus reuniens [72,73] and medial dorsal thalamic nucleus [74]. In turn, all of these areas send projections to the medial prefrontal cortex [70]. Similarly, projections arising from the mPFC are returned to the HC via multiple pathways: including a recently described direct connection from mPFC to dorsal CA1 [75] via the entorhinal cortex [76] through the nucleus reuniens [72,73] and via the medial dorsal thalamic nuclei ([74]; see Figure 3). Some or all of these multiple avenues might be the facilitators for the important interactions between the HC and mPFC that occur during learning and memory.

One of the main curiosities of mPFC-HC theta interaction research is the apparent disconnect between the anatomical connections and electrophysiology. As discussed above, only the ventral areas of the HC directly project to the mPFC; however, most electrophysiological studies record in the dorsal HC. While neurons and field potentials in the mPFC are synchronized with theta rhythms recorded in both the dorsal and ventral aspects of the HC [1], more detailed observations and analysis have shown that neural synchrony between the mPFC and HC is likely mediated by ventral regions [77,78]. Thus, it would be reasonable to surmise that mPFC-HC theta interactions are the product of activation of the direct excitatory projections from ventral CA1 to the mPFC. In conjunction, the temporal offset between mPFC unit maximum phase-locking [1] is nearly identical to the conduction delay along this ventral CA1 to the mPFC pathway [4,76,77,78]. A recent study by Hallock and colleagues [79] offers a confounding result that directly questions this conclusion, and instead suggests that connections routed through the nucleus reuniens are of primary importance. They recorded LFPs in mPFC and HC while they inactivated the nucleus reuniens and found that both theta synchrony and spatial working memory performance decreased. It should also be noted that there are dense projections from the nucleus reuniens to ventral CA1 and that the same reuniens neurons project to both ventral CA1 and the mPFC, so these findings are not entirely inconsistent with the conclusions of O’Neill and colleagues [80]. Clearly, there is still much to be learned about the relationship between the complex anatomical connections between the mPFC and the HC and how they are involved in both electrical and spatial learning interactions. It is possible that theta interactions either can arise via different pathways connecting the two areas or might involve the simultaneous activation of multiple pathways, or might be mediated by a different structure simultaneously entraining both the HC and mPFC. At this point, it is unclear which is correct, and it is certainly possible that there are multiple ways for theta interactions to emerge; however, more research is needed into this important question. 

Another way to approach the problem is to examine for functional differences between the dorsal and ventral HC. While it is true that both areas are involved in spatial learning [81,82] and spatial working memory [83], more robust spatial deficits are found following dorsal insults/inactivations [84,85] while ventral insults/inactivations primarily impair olfactory working memory [86] and contextual and trace fear conditioning [87,88,89]. These studies have supported the longstanding idea that the dorsal HC is more involved in spatial navigation and learning, while the ventral areas are more important for emotion and olfaction [90]. While these lesion studies make a compelling case for a functional topography along the longitudinal axis of the HC, the differences revealed in unit recordings are not as robust. Multiple reports have shown that there is a systematic increase in the place field size from the dorsal to the ventral pole [91,92,93], but it is not clear whether this amounts to any real differences in spatial information processing. Keinath and colleagues [94] examined spatial representations along this axis and found no substantive differences in terms of cue or context-induced remapping in neural spatial reconstruction at the population-level. While more research is needed to more clearly understand the different roles the dorsal and ventral HC, the idea that there is a hard disassociation where spatial information is only present in dorsal CA1 is not accurate. Many of these questions are outside the scope of the current review; however, in terms of mPFC-HC theta interactions, does it really matter if recordings are made in dorsal or ventral HC? The available data seem to indicate it does not, as Jones and Wilson [95] found no distinguishable correlations in how dorsal or ventral CA1 neurons were coactive with mPFC neurons during spatial memory tasks. 

### 3.2. HC-mPFC Interactions during Spatial Processing

To better understand the relevance of theta interactions between brain areas, Siapas et al. [1] recorded hippocampal and mPFC neurons and local field potentials (LFPs) during an array of spatial tasks. The data from these experiments resulted in the first reported evidence that mPFC neurons are phase-locked to hippocampally generated theta rhythms. Additionally, it was revealed that this entrainment occurs during multiple behavioral paradigms including: the eight-arm radial maze, spatial delayed alternation, and exploration of novel environments. Because this result was found during multiple behaviors, it suggests that phase-locking was not merely the product of spatial working memory calculations, but rather may have reflected a more general processing of spatial information. However, the practical relevance of this finding was not made apparent until a number of other studies examined how this type of communication relates to more specific types of behavior. Our lab recorded mPFC units and hippocampal LFPs while animals performed a spatial delayed non-match to sample task, which required subjects to perform a sequenced chain of behaviors: lever press, nose poke, and an additional non-matching lever press to receive a reward. We found that, in trials, animals made errors and there was a huge drop-off in the number of mPFC neurons that were phase-locked to the hippocampal theta rhythm [3]. Interestingly, this occurred equally during both the sample and test phases, which made it impossible to determine if these theta interactions were important for encoding or retrieving the sample stimulus information. Similarly, Jones and Wilson [95] found that that in erroneous delayed alternation trials, mPFC-HC theta phase-locking and coherence were significantly lower. Furthermore, they also found that both of these metrics were strongest at the choice point in a T-maze, which again made it difficult to discern exactly what information was being communicated between areas. In this case, the information could have been prospective (“go right ahead”) or retrospective (“went left last trial”); nonetheless, it was clear that strong theta interactions were associated with task accuracy. 

We have seen evidence that synchrony between the HC and mPFC is important for spatially dependent and working memory behaviors, although implications beyond a mere flow of information have remained elusive. Benchenane and colleagues [4] revealed another role for mPFC-HC theta interactions in influencing the formation of functional neural networks. After subjects were fully trained on a dual rule y-maze task, the researchers switched the allocentric cues, which required the subjects to relearn task rules. During this ‘relearning’ phase, it was shown that more mPFC neurons were entrained to theta and that mPFC cell assemblies were formed during periods with high theta coherence. Altogether, these results have shown that working memory task learning and performance is directly influenced by the strength of theta frequency interactions between these two areas. This point was conclusively shown in 2016, when researchers inactivated the nucleus reuniens while simultaneously recording in both the mPFC and HC. This inactivation prevented mPFC-HC theta synchrony and also impaired working memory performance [80]. Future work will hopefully be able to provide insights about the specific nature of the information being conveyed between the HC and mPFC during working memory tasks (i.e., retrospective/prospective or encoding/retrieval or attention-related), but, at this point, there is a substantial amount of correlative data that indicate that theta interactions between these areas somehow enable successful spatial working memory performance.

### 3.3. Prefrontal Influence on HC Function

The above results lead to two equally reasonable possibilities about working memory theta interactions: (1) they represent the sharing of some types of trial specific task information (i.e., the last trial was a right turn or the sample was the left lever); (2) they are indicative of enhanced information transfer in a more general sense. This second possibility more readily addresses non-working memory tasks, where it is difficult to postulate in any way about what information is being shared between the HC and mPFC. Our own lab has found strong mPFC unit phase-locking to hippocampal theta rhythm in non-working memory spatial tasks (environment exploration, alleyway maze) [96], and these mPFC-HC interactions are mechanistically similar to the working memory findings, in that each case involves theta frequency synchronous unit firing in the two areas. Other work has revealed that the relationship between these two areas is more complex and nuanced than the mere sharing of working memory information. In a series of experiments, Kyd and Bilkey studied the effects of mPFC lesions on hippocampal place cell activity, finding that CA1 place fields were abnormally sized, less stable over time, and more reactive to changes in the local environment than in undisturbed animals [97,98]. Another lab found that mPFC inactivation did not affect previously formed CA1 place fields at a goal area, but did overall make place cell activity less variable [99]. It has previously been suggested that firing variance within a place field is indicative of various cognitive aspects of spatial processing, including the possible switching between different spatial codes that is necessary during alternation tasks [13,100], and this is argued to be a measure of cognitive flexibility [101,102] It appears that mPFC activity is helping to modulate hippocampal place cell firing variance and, in turn, may affect the flexible usage of spatial information. It is possible that the more contextual spatial signal found in mPFC ensembles helps to serve as a “unifying” behaviorally-based message for hippocampal place cells. This could help to make place cells less likely to be at the whim of changes to local cues rather than cognitive cues like a ‘goal area’ and also allow for the flexible usage of multiple spatial codes depending on behavioral needs. Thus, the mPFC might be important for helping to make the CA1 place code more adaptable to keep up with changing cognitive demands. 

## 4. Dependency Shift after Consolidation

Scoville and Milner [103] were the first to report the hippocampus’ importance for forming new declarative memories in their landmark case study of Henry Molaison. However, this area is not solely responsible for storing and retrieving past experiences; rather, it mediates interactions with surrounding brain areas [104] which enables temporal reorganization [105,106] and facilitates long-term memory storage. Extrahippocampal memory storage has proven to be quite complex and includes many separate brain areas; for example, the cerebellum [107], entorhinal cortex [108], auditory cortex [109], visual associations areas [110], and prefrontal areas [5] have all been implicated in some type of memory storage. 

A central problem when studying long-term memories is being able to differentiate between memory storage and retrieval. Memory storage generally refers to structural changes at the synaptic level while retrieval can be seen in the pattern of activity across a neuronal ensemble or the successful usage of previously learned information [111]. Certainly, these processes are for the most part directly related since stronger synapses between cells will make those same cells more likely to be co-activated [112]. The tight relationship between these two processes is illuminated by administering compounds such as anisomycin that disrupt long-term structural changes to synapses either during or shortly after learning. These compounds effectively disable memory storage and subsequently impair memory retrieval [113,114,115]. Conversely, retrieval can be disrupted by inactivating neurons during recall sessions, as detailed in multiple studies discussed below. However, disentangling these two processes has remained difficult, but, recently, methodological innovations have made it possible to experimentally differentiate between these two processes, helping to parse out the clear distinction that exists between memory storage and retrieval. By optogenetically-inducing long-term depression in amygdalar neurons, and thus disrupting the storage mechanism of the memory trace, Nabavi and colleagues were able to prevent the retrieval of that memory; however, when the same neural network was optogenetically-excited, the fear behavior returned [116]. Thus, the authors were able to show that memory retrieval is possible even after disrupting memory storage. While conceptually and mechanistically linked, these two processes can be disassociated with careful experimentation; however, in most studies, it is not possible to separate out these processes. Further complicating the literature, researchers use the term engram or trace to refer to both the active cells that make up a memory retrieval ensemble and the synaptically connected network of cells that make up memory storage. In this paper, we use the term engram to refer to a specific pattern of activity across a neuronal population that is activated during memory encoding and retrieval. In this section, we will first review studies into the long-term storage of navigational and contextual information within the mPFC, then highlight possible mechanisms for memory storage and readout, and, lastly, examine shifts in activity states during remote memory recall.

### 4.1. Evidence from Pharmacological Inactivation

For many creatures, the ability to memorize the locations of reliable food sources to exploit, while also avoiding areas of high predation, is critical for their survival. It has previously been shown that inactivation of the mPFC disrupts retrieval of remotely formed conditioned fear memories [6], conditioned taste aversion [117], spatial learning [5], and spatial navigation [15]. Such results readily invite the conclusion that these memories have become completely reliant on prefrontal areas [118]. An early example of this mechanism was discovered with a series of closed field maze tests. It was found that posterior cingulate cortex lesions only affect recall if the damage occurs shortly after initial training (11 days); however, ACC lesions affect performance when they are made long after acquisition (48 days; [119]. Another example of the ACC’s involvement in remote memory recall comes from Liu and colleagues [120], who trained rodents on an inhibitory avoidance task employing contextual cues and foot shocks. After three consecutive days of training, the ACC was inactivated and subjects were retested at a later time. Subjects in the ACC lesion group did not exhibit any avoidance to the conditioning chamber, which suggests that the ACC has an important role for recollection of a consolidated fear memory. These findings show that the mPFC, including the ACC, is an important brain structure for using remote memories to perform spatial navigation tasks, but they do little in explaining the mechanisms at work during recollection or the nature of the memory trace that may persist in the mPFC long after initial learning. 

### 4.2. Electrophysiological Evidence

Memory traces, like those found in the HC [28], may exist in the mPFC, though, at this point, the evidence is merely suggestive. Neuronal firing patterns that are consistent with memory usage in this area are implicated in a wide array of tasks including foraging [121], anticipation [122,123], error detection [124], avoidance [120], and consolidation [125]. An integral part of memory formation is believed to be temporally compressed replay of recently experienced neuronal firing sequences during sleep [126]. Though this process was first characterized in the HC, mPFC cells also exhibit accelerated replay of previously learned tasks [127] and rules [128]. Compressed replay is widely believed to be an important aspect of long-term memory formation and storage, by helping to engender neuronal plasticity [129,130,131]. During learning, there are dynamic changes in prefrontal networks that coincide with behavioral improvement [132] and elevated mPFC activity during learning is predictive of later recollection [133]. In addition, throughout learning of context-dependent associations, firing patterns in mPFC evolve until maximal learning has taken place and they become fixed [134,106]. Dynamic shifts in mPFC activity throughout learning of new memories and retrieval of existing memories suggest that this area plays an integral role in memory processes [135]. In each of these findings, we see mPFC neurons displaying, or undergoing, a process that is similar to processes reported in hippocampal neurons that most would argue are indicative of memory formation [16]. In addition, these findings may explain why such profound deficits were observed in spatial navigation and contextual memories resulting from mPFC inactivation [15]. Taken together, it is hard to argue against the importance of the mPFC for completion of a remotely learned task. It is certainly possible that, throughout the learning and consolidation processes, areas including the mPFC are encoding and storing information about experiences. However, it should be noted that there have been no reports of human cingulotomy patients encountering long-term memory problems [136]. It is possible that this is due to an inherent difference between species that has yet to be isolated. However, it is also possible that the nature of the information stored in the mPFC trace may not be what is implied by findings showing behavioral failures in remote memory spatial navigation tasks. We will revisit this idea in a section below, but, for now, we can safely conclude that currently there is strong evidence supporting the role of prefrontal areas in some aspect of memory storage and recollection in rodents.

### 4.3. Activity States during Remote Memory Recall

A better understanding of activity states in the mPFC during memory readout may help to explain the neural processes for memory recollection. Among this area of study, experiments utilizing immediate early genes (IEG) and optogenetics have made remarkable strides in outlining physical characteristics of remote memory recall. Utilizing a notable IEG, c-fos, researchers have been able to map out large cortical networks and identify memory specific connectomes [137]. Furthermore, utilizing a combination of optogenetics and c-fos researchers has identified hippocampal networks relating to spatial memories, and, through stimulation, has been able to induce false recollection [28]. Importantly, c-fos expression is a powerful metric for examining how and what brain areas are activated during recollection of remotely formed memories. Frankland and colleagues [6] examined how mPFC activity changed as memories progress from being recently formed to more remote. They conditioned mice to fear environmental cues until maximum learning had taken place, and it was found that c-fos expression was elevated in the ACC after consolidation had occurred. Separately, subjects were given lidocaine injections to the ACC at either 1, 3, 18, or 36 days after learning. Subjects in the one and three-day groups exhibited freezing behavior much like controls, whereas subjects in the 18 and 36 day conditions displayed significantly less freezing behavior. This result tells us that the mPFC is inherently active, while recalling contextual information about past experiences, but only after a set amount of time. When considered together with their c-fos findings, these results suggest not only that the ACC is important for remote memory recall, but also that the memory itself may be stored in ACC neurons. This result was later replicated with a different behavioral paradigm, when Teixeira et al. [138] used a water maze to test recall of a specific location during recent (one-day) or remote (30 day) retrieval. The researchers measured c-fos expression in the ACC during recent or remote trial conditions, and it was again found that the ACC exhibits elevated activity during the remote recall phase. Next, researchers utilized pharmacological inactivation of either the ACC or HC and found, unsurprisingly, that animals with recent or remote hippocampal inactivation were impaired; however, ACC inactivation only impaired recall in the remote condition. While lesion/inactivation studies alone can identify brain areas important for some function, they leave open the possibility that the lesioned area might only be serving as a pathway or throughput for information that is needed by other important areas. By pairing lesion/inactivation results with cellular activity markers, this research has established that the important neuronal processes for remote spatial memory usage are indeed occurring within the ACC. This idea was further established with the aid of optogenetic stimulation. Mice were trained to fear foot shocks associated with specific environments while also habituating to other environments. Researchers first genetically tagged ACC neurons that were active in the shock environment. Then, they optogenetically excited these ACC neurons in a neutral environment and thus induced conditioned fear memories as seen through elevated freezing [74]. These results, and the others discussed here, directly support the idea that ACC neurons are of primary importance for forming and retrieving spatial memories. 

## 5. Ideas on the Nature of the mPFC Memory Trace

The findings detailed in this paper make a very strong case that, at least in the rodent, the mPFC is an integral area for the usage of remote spatial memories. However, what information exactly is contained in a consolidated mPFC memory trace is still far from certain. Some of the most influential theories about the mPFC’s role in long-term memory posit that, after time has passed, the mPFC plays a role during remote recall that is inherently similar to the role the HC plays during recent recall. That is, the mPFC helps to coordinate activity in diverse sets of neurons in various cortical association areas where the individual aspects of the memory are thought to be stored [139,140]. Theoretically there are, at least, two distinct possibilities for what this might mean about an mPFC memory trace: (1) it is inherently similar to the HC trace due to the HC passing on its trace to the mPFC over the course of consolidation; or (2) it is unique from the HC trace, but the mPFC trace is more durable, and, after, the HC trace naturally fades over time, the mPFC trace is all that remains. In both situations, after time has passed, memory usage will be dependent on the mPFC; however, the actual nature of the information in the mPFC should be different. The first characterization suggests that mPFC activity should be rather similar to hippocampal activity, and in some cases that are thought to be strongly related to memory processing, this is true; for example, neurons in both areas exhibit strong stimulus and response behavioral correlates [141,142]; exhibit theta phase precession [95]; and show compressed replay of neuronal activity [127]. Notably absent, however, from any similarities are detailed spatial signals, which interestingly are precisely the type of remote memories first found to be mPFC dependent [5]. While the experimental evidence certainly supports a substantial role for the mPFC in remote memory readout, it is clear that the mPFC signal is not merely a mirror image of the hippocampal signal. The mPFC spatial signal is highly distributed and only manifests when whole ensembles are considered [19], but the HC contains a strikingly sparse spatial code ([143]; see Figure 1 and Figure 2). Notice how, in Figure 2, as more mPFC neurons are included in the analysis, more spatial information represented across the ensemble, while, for hippocampal ensembles, less information is gained by considering more neurons. This is certainly due to inherent structural differences between the two areas, but it also is likely indicative of unique processes occurring in each area. The second possibility introduced above posits that the mPFC is forming an alternative mnemonic representation in parallel to the HC, and that, in essence, both traces are quite different. Working memory studies show that the two areas are freely sharing information and it is also clear that activity in each area can affect processing in the other, which would suggest separate but complementary processes.

If the mPFC is not storing hippocampal-like information, such as a place code, then what information is stored in the mPFC to begin with? Another way to address this point is to ask what unique signal is the mPFC contributing to for hippocampally-formed memories? We believe that the answer to these questions can be found by examining the results from electrophysiological recordings from the mPFC and HC, or, rather, to understand storage, we need to examine retrieval activity, which hypothetically should be inherently similar to encoding activity. We know that single mPFC cells contain very little spatial information except for areas around a goal location [19,120]; however, across mPFC ensembles, a clear contextual signal appears that incorporates spatial information with behavioral relevance [19,44,45,46,144,145,146]. In fact, the larger the number of mPFC neurons considered, the greater the amount of contextual information ([19,144]; see Figure 2). This highly distributed contextual signal provides a framework for incorporating behavioral with spatial information and maybe this is exactly what the mPFC is initially passing on to the HC, and, in turn, maybe this framework is what the HC is consolidating back out to the mPFC, or this signal just persists after the HC trace has naturally faded. Thus, remote memory usage failures occur when the mPFC is inactivated because this framework is missing from the retrieved hippocampal trace. Furthermore, the mPFC signal might be structured based upon spatial information, such as in the absence of an overt task, or it may be influenced by cognitive/behavioral information. This provides the mPFC signal with a high degree of flexibility, which is in line with the mPFC’s substantial role in behavioral flexibility [147,148,149,150,151,152].

**Figure 4:**
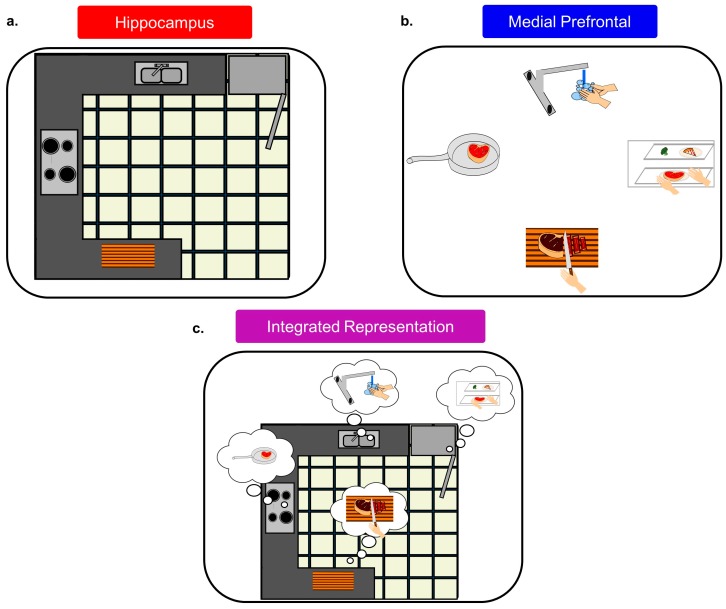
Schematic of the unique coding properties of the hippocampus (HC) and medial prefrontal cortex (mPFC), and their integration. (**a**) purely spatial coding in HC; (**b**) behaviorally-driven coding in mPFC; (**c**) in the integrated representation, the appropriate behaviors are associated with locations.

The mPFC context signal could serve as a lattice for the internal hippocampal representation, allowing certain locations/stimuli to be associated with behavioral relevance (see Figure 4). This would readily explain why Hok et al. [99] found evidence of decreased cognitive flexibility in HC circuits after mPFC inactivation, and it would also explain why Kyd and Bilkey [97,98] found that mPFC inactivation made CA1 place cells more likely to be influenced by local cues. Thus, the mPFC serves to help unify information into a singular context signal and also allows for behavioral, cognitive, and emotional information to be attached to specific locations/responses/stimuli that may appear within this context. In fact, mPFC cells have been found to “tag” locations in working memory, tracking how often they had been visited [146] similar to how mPFC ensembles ‘tag’ consecutive operant responses during a sequence chain [153]. Both of these findings are consistent with the mPFC being integral for incorporating spatial with behavioral information into a singular representation or context signal.

A “unifying” framework, or context signal, also explains why mPFC lesions only impair performance when tasks become more difficult and require behavioral flexibility [154,155,156]. To flexibly respond in a situation where only partial information is given or when there is a shift in rules requires one to incorporate multiple possible responses within a unified framework. For instance, when de Bruin et al. [147] challenged mPFC lesioned animals with a water maze reversal, these animals failed to rapidly find the new goal location; however, these same animals had no deficits when initially learning the first goal location. In this case, the mPFC would be providing a behavioral framework of how the task works, such as: get dropped in water and find a safe location. It is possible animals that with lesioned mPFCs were able to learn the task by simply memorizing one location for safety, while still not conceptualizing the larger idea that safe locations exist and finding them is part of the task. Consistent with this interpretation, Granon and Poucet [157] found that mPFC inactivation did not impair water maze performance when novel start sites were used for the same goal location. Completing this type of task requires incorporating spatial information with behavioral and cognitive information to form a rule representation. Similarly, one could imagine that, in alternation tasks, the mPFC is helping with the idea that the goal location switches locations between trials. This requires the animal to flexibly switch between hippocampal space codes to get to the correct goal on consecutive trials [158], and mPFC lesions do, in fact, impair hippocampal place code flexibility [99]. In many ways, all of these tasks and processes require the animal to consider individual elements (locations, responses) while simultaneously being aware of all the other possibilities, and one way to accomplish this would be to have each element also linked to a larger context framework. Thus, when any single item is considered, the context framework is primed and thus any other items associated with that context framework would be readily available. This would explain why mPFC response and stimulus representations are distinct depending on which context (either environmental or behavioral) the animal is in [19,44,46]. 

As to what information is consolidated to the mPFC, well, when consolidation is discussed in other brain areas, it is widely considered that the originally encoded information is what is eventually permanently stored, even across the same exact neural networks [109,159]. Why would one believe that the mPFC would be any different? Thus, animals fail to accurately navigate to a goal location after long retention intervals if the mPFC is inactivated, not because they have forgotten the maze or the room the maze was in, but rather they have lost the behavioral framework within which to utilize the spatial information. This is similar to the conceptualization posited by Weible [160], but instead of his strong emphasis on attention, we put forward that the mPFC is providing a framework to incorporate all sorts of cognitive processes (emotional valence, rules, and sequences) with spatial information, and this framework is what will eventually be stored within the mPFC. It is equally possible that since the encoded mPFC trace never contained specific spatial information aside from the goal location, that with mPFC taken offline for remote recall, the animal might still be aware of its location within an environment, but there is no memory of what makes the goal location different from all other locations. All of these possibilities are based upon observations from mPFC recording studies, and thus the answer to what is consolidated to the mPFC has already been answered, and is evidenced in every mPFC electrophysiological study. 

This idea leads to several readily testable predictions. For example, when the mPFC is taken offline for remote retrieval, all the individual aspects of a remembered memory would be present in other brain areas, but without the mPFC’s framework for this information, none of the locations/responses/stimuli would have much meaning. Thus, in this situation, if one was to record from area CA1, one could expect to see relatively intact hippocampal place codes even though the animal might still fail to find the correct goal location. In remote recall of conditioned fear, CA1 place fields should again be intact even though freezing might not occur. In a task that requires the mPFC, such as set-shifting, one would expect the mPFC to lead the HC when the animal switches between rules. Similarly, when an animal approaches a goal location, the mPFC should, at least momentarily, lead the HC as well. Indeed, Place et al. [161] found that when animals explored objects within a known environment, the mPFC oscillated first and the HC was entrained to the mPFC’s theta rhythm. Evidence can also be seen when mPFC neurons fire during remote recall to locations that had previously had an object placed there [162]. Additionally, if the HC trace naturally fades and the mPFC trace persists, in any remote recall situation, we might expect the mPFC to retrieve the memory without any change in hippocampal activity. Indeed, if the retrieved mPFC context signal is being utilized by the HC to activate the more spatially specific representation present in hippocampal networks, then the mPFC should take the lead and hippocampal activity should be entrained to mPFC oscillations. While none of these individual predictions, if proven, would completely confirm this idea, together they would provide a better understanding of what exactly is stored in the mPFC and allow future debates to concentrate on the more esoteric details such as the relative importance of attention as opposed to emotion, cognition, or context.

## 6. Conclusions

In this review, we have examined recent findings that relate to the importance of interactions between the HC and mPFC for processing and storing spatial information. We have discussed evidence supporting the idea that both the mPFC and HC process spatial information in unique ways. We have shown that, during the completion of many different types of spatial tasks, interactions between these two brain areas mediate behaviors and accompany successful execution. Lastly, we have looked at shifts in memory dependency throughout consolidation. While it is quite clear that interactions between the mPFC and HC are important for successful execution of many behaviors and other cognitive processes, there are still a number of questions that have not yet been answered: what type of information is being transmitted between brain areas during spatial working memory tasks? Does the mPFC store memory traces that help to mediate behaviorally relevant recall in the HC? Are spatial working memory and consolidation a related process? Is the mPFC forming and storing a unique representation of experience independent from the HC? Clearly, after a certain amount of time has passed, executing tasks demanding the retrieval of a contextual memory requires an intact mPFC, but knowing what aspects of that memory trace have been stored there remains elusive.

## References

[1] Siapas A.G., Lubenov E.V., Wilson M.A. (2005). Prefrontal phase locking to hippocampal theta oscillations. Neuron.

[2] Jones M.W., Wilson M.A. (2005). Theta rhythms coordinate hippocampal–prefrontal interactions in a spatial memory task. PLoS Boil..

[3] Hyman J.M., Zilli E.A., Paley A.M., Hasselmo M.E. (2010). Working memory performance correlates with prefrontal-hippocampal theta interactions but not with prefrontal neuron firing rates. Front. Integr. Neurosci..

[4] Benchenane K., Peyrache A., Khamassi M., Tierney P.L., Gioanni Y., Battaglia F.P., Wiener S.I. (2010). Coherent theta oscillations and reorganization of spike timing in the hippocampal-prefrontal network upon learning. Neuron.

[5] Bontempi B., Laurent-Demir C., Destrade C., Jaffard R. (1999). Time-dependent reorganization of brain circuitry underlying long-term memory storage. Nature.

[6] Frankland P.W., Bontempi B., Talton L.E., Kaczmarek L., Silva A.J. (2004). The involvement of the anterior cingulate cortex in remote contextual fear memory. Science.

[7] Howard M.W., Fotedar M.S., Datey A.V., Hasselmo M.E. (2005). The Temporal Context Model in spatial navigation and relational learning: Toward a common explanation of medial temporal lobe function across domains. Psychol. Rev..

[8] Morris R.G.M., Garrud P., Rawlins J.N.P., O’Keefe J. (1982). Place navigation impaired in rats with hippocampal lesions. Nature.

[9] O’Keefe J., Dostrovsky J. (1971). The hippocampus as a spatial map. Preliminary evidence from unit activity in the freely-moving rat. Brain Res..

[10] Sanders H., Rennó-Costa C., Idiart M., Lisman J. (2015). Grid Cells and Place Cells: An Integrated View of their Navigational and Memory Function. Trends Neurosci..

[11] Pastalkova E., Itskov V., Amarasingham A., Buzsáki G. (2008). Internally Generated Cell Assembly Sequences in the Rat Hippocampus. Science (New York, N.Y.).

[12] Wood E.R., Dudchenko P.A., Eichenbaum H. (1999). The global record of memory in hippocampal neuronal activity. Nature.

[13] Wood E.R., Dudchenko P.A., Robitsek R.J., Eichenbaum H. (2000). Hippocampal neurons encode information about different types of memory episodes occurring in the same location. Neuron.

[14] Griffin A.L., Eichenbaum H., Hasselmo M.E. (2007). Spatial representations of hippocampal CA1 neurons are modulated by behavioral context in a hippocampus-dependent memory task. J. Neurosci..

[15] Seamans J.K., Floresco S.B., Phillips A.G. (1995). Functional differences between the prelimbic and anterior cingulate regions of the rat prefrontal cortex. Behav. Neurosci..

[16] Buzsáki G., Moser E.I. (2013). Memory, navigation and theta rhythm in the hippocampal-entorhinal system. Nat. Neurosci..

[17] Tsien J.Z., Huerta P.T., Tonegawa S. (1996). The essential role of hippocampal CA1 NMDA receptor–dependent synaptic plasticity in spatial memory. Cell.

[18] Broadbent N.J., Squire L.R., Clark R.E. (2004). Spatial memory, recognition memory, and the hippocampus. Proc. Natl. Acad. Sci. USA.

[19] Hyman J.M., Ma L., Balaguer-Ballester E., Durstewitz D., Seamans J.K. (2012). Contextual encoding by ensembles of medial prefrontal cortex neurons. Proc. Natl. Acad. Sci. USA.

[20] McNamara C.G., Tejero-Cantero Á., Trouche S., Campo-Urriza N., Dupret D. (2014). Dopaminergic neurons promote hippocampal reactivation and spatial memory persistence. Nat. Neurosci..

[21] Lasztóczi B., Klausberger T. (2016). Hippocampal Place Cells Couple to Three Different Gamma Oscillations during Place Field Traversal. Neuron.

[22] O’keefe J., Conway D.H. (1978). Hippocampal place units in the freely moving rat: Why they fire where they fire. Exp. Brain Res..

[23] Eichenbaum H., Dudchenko P., Wood E., Shapiro M., Tanila H. (1999). The hippocampus, memory, and place cells: Is it spatial memory or a memory space?. Neuron.

[24] Foster D.J., Wilson M.A. (2006). Reverse replay of behavioural sequences in hippocampal place cells during the awake state. Nature.

[25] Leutgeb S., Leutgeb J.K., Barnes C.A., Moser E.I., McNaughton B.L., Moser M.B. (2005). Independent codes for spatial and episodic memory in hippocampal neuronal ensembles. Science.

[26] Alme C.B., Miao C., Jezek K., Treves A., Moser E.I., Moser M.B. (2014). Place cells in the hippocampus: Eleven maps for eleven rooms. Proc. Natl. Acad. Sci. USA.

[27] Jezek K., Henriksen E.J., Treves A., Moser E.I., Moser M.B. (2011). Theta-paced flickering between place-cell maps in the hippocampus. Nature.

[28] Ramirez S., Liu X., Lin P.A., Suh J., Pignatelli M., Redondo R.L., Tonegawa S. (2013). Creating a false memory in the hippocampus. Science.

[29] Bizley J.K., King A.J. (2008). Visual–auditory spatial processing in auditory cortical neurons. Brain Res..

[30] Watanabe J., Sugiura M., Miura N., Watanabe Y., Maeda Y., Matsue Y., Kawashima R. (2004). The human parietal cortex is involved in spatial processing of tongue movement—an fMRI study. Neuroimage.

[31] Rauschecker J.P., Tian B. (2000). Mechanisms and streams for processing of “what” and “where” in auditory cortex. Proc. Natl. Acad. Sci. USA.

[32] Peck E.L., Peck C.J., Salzman C.D. (2014). Task-Dependent Spatial Selectivity in the Primate Amygdala. J. Neurosci..

[33] Pych J.C., Chang Q., Colon-Rivera C., Haag R., Gold P.E. (2005). Acetylcholine release in the hippocampus and striatum during place and response training. Learn. Mem..

[34] Horst N.K., Laubach M. (2012). Working with memory: Evidence for a role for the medial prefrontal cortex in performance monitoring during spatial delayed alternation. J. Neurophysiol..

[35] Chang D., Lissek S., Ernst T.M., Thürling M., Uengoer M., Tegenthoff M., Timmann D. (2015). Cerebellar contribution to context processing in extinction learning and recall. Cerebellum.

[36] Zelikowsky M., Hersman S., Chawla M.K., Barnes C.A., Fanselow M.S. (2014). Neuronal Ensembles in Amygdala, Hippocampus, and Prefrontal Cortex Track Differential Components of Contextual Fear. J. Neurosci..

[37] Roozendaal B., Portillo-Marquez G., McGaugh J.L. (1996). Basolateral amygdala lesions block glucocorticoid-induced modulation of memory for spatial learning. Behav. Neurosci..

[38] Custódio D.S., Cardoso J., Martins C.W., Lugon M.D., Fregni F., Nakamura-Palacios E.M. (2013). Epidural direct current stimulation over the left medial prefrontal cortex facilitates spatial working memory performance in rats. Brain Stimul..

[39] Yang S.-T., Shi Y., Wang Q., Peng J.-Y., Li B.-M. (2014). Neuronal representation of working memory in the medial prefrontal cortex of rats. Mol. Brain.

[40] Floresco S.B., Seamans J.K., Phillips A.G. (1997). Selective roles for hippocampal, prefrontal cortical, and ventral striatal circuits in radial-arm maze tasks with or without a delay. J. Neurosci..

[41] Song C., Ehlers V.L., Moyer J.R. (2015). Trace Fear Conditioning Differentially Modulates Intrinsic Excitability of Medial Prefrontal Cortex–Basolateral Complex of Amygdala Projection Neurons in Infralimbic and Prelimbic Cortices. J. Neurosci..

[42] Euston D.R., McNaughton B.L. (2006). Apparent encoding of sequential context in rat medial prefrontal cortex is accounted for by behavioral variability. J. Neurosci..

[43] Poucet B., Chaillan F., Truchet B., Save E., Sargolini F., Hok V. (2015). Is there a pilot in the brain? Contribution of the self-positioning system to spatial navigation. Front. Behav. Neurosci..

[44] Ma L., Hyman J.M., Durstewitz D., Phillips A.G., Seamans J.K. (2016). A Quantitative Analysis of Context-Dependent Remapping of Medial Frontal Cortex Neurons and Ensembles. J. Neurosci..

[45] Lapish C.C., Durstewitz D., Chandler L.J., Seamans J.K. (2008). Successful choice behavior is associated with distinct and coherent network states in anterior cingulate cortex. Proc. Natl. Acad. Sci. USA.

[46] Durstewitz D., Seamans J.K. (2008). The dual-state theory of prefrontal cortex dopamine function with relevance to catechol-o-methyltransferase genotypes and schizophrenia. Biol. Psychiatry.

[47] Caracheo B.F., Emberly E., Hadizadeh S., Hyman J.M., Seamans J.K. (2013). Abrupt changes in the patterns and complexity of anterior cingulate cortex activity when food is introduced into an environment. Front. Neurosci..

[48] Reverberi C., Görgen K., Haynes J.D. (2012). Compositionality of rule representations in human prefrontal cortex. Cereb. Cortex.

[49] Gray C.M. (1994). Synchronous oscillations in neuronal systems: Mechanisms and functions. J. Comput. Neurosci..

[50] Winson J. (1978). Loss of hippocampal theta rhythm results in spatial memory deficit in the rat. Science.

[51] Larson J., Wong D., Lynch G. (1986). Patterned stimulation at the theta frequency is optimal for the induction of hippocampal long-term potentiation. Brain Res..

[52] O’Keefe J. (1993). Hippocampus, theta, and spatial memory. Curr. Opin. Neurobiol..

[53] Hyman J.M., Wyble B.P., Goyal V., Rossi C.A., Hasselmo M.E. (2003). Stimulation in hippocampal region CA1 in behaving rats yields long-term potentiation when delivered to the peak of theta and long-term depression when delivered to the trough. J. Neurosci..

[54] Green J.D., Arduini A.A. (1954). Hippocampal electrical activity in arousal. J. Neurophysiol..

[55] Vanderwolf C.H., Bland B.H., Whishaw I.Q. (1973). Diencephalic, Hippocampal, and Neocortical Mechanisms in Voluntary Movement.

[56] McFarland W.L., Teitelbaum H., Hedges E.K. (1975). Relationship between hippocampal theta activity and running speed in the rat. J. Comp. Physiol. Psychol..

[57] Macrides F., Eichenbaum H.B., Forbes W.B. (1982). Temporal relationship between sniffing and the limbic theta rhythm during odor discrimination reversal learning. J. Neurosci..

[58] Vanderwolf C.H., Kramis R., Gillespie L.A., Bland B.H. (1975). Hippocampal Rhythmic Slow Activity and Neocortical Low-Voltage Fast Activity: Relations to Behavior. The Hippocampus.

[59] Grastyan E., Karmos G., Vereczkey L., Kellenyi L. (1966). The hippocampal electrical correlates of the homeostatic regulation of motivation. Electroencephalogr. Clin. Neurophysiol..

[60] Winson J. (1974). Patterns of hippocampal theta rhythm in the freely moving rat. Electroencephalogr. Clin. Neurophysiol..

[61] Buzsáki G. (2002). Theta oscillations in the hippocampus. Neuron.

[62] Seidenbecher T., Laxmi T.R., Stork O., Pape H.C. (2003). Amygdalar and hippocampal theta rhythm synchronization during fear memory retrieval. Science.

[63] Hoffmann L.C., Berry S.D. (2009). Cerebellar theta oscillations are synchronized during hippocampal theta-contingent trace conditioning. Proc. Natl. Acad. Sci. USA.

[64] Albertin S.V., Wiener S.I. (2015). Neuronal Activity in the Nucleus Accumbens and Hippocampus in Rats during Formation of Seeking Behavior in a Radial Maze. Bull. Exp. Biol. Med..

[65] Cai D., Aharoni D., Shuman T., Shobe J., Biane J., Song W., Silva A. (2016). A shared neural ensemble links distinct contextual memories encoded close in time. Nature.

[66] Xu W., Südhof T.C. (2013). A neural circuit for memory specificity and generalization. Science.

[67] Lopez J., Herbeaux K., Cosquer B., Engeln M., Muller C., Lazarus C., de Vasconcelos A.P. (2012). Context-dependent modulation of hippocampal and cortical recruitment during remote spatial memory retrieval. Hippocampus.

[68] Swanson L.W. (1981). A direct projection from Ammon’s horn to prefrontal cortex in the rat. Brain Res..

[69] Vertes R.P. (2006). Interactions among the medial prefrontal cortex, hippocampus and midline thalamus in emotional and cognitive processing in the rat. Neuroscience.

[70] Amaral D.G., Witter M.P. (1989). The three-dimensional organization of the hippocampal formation: A review of anatomical data. Neuroscience.

[71] Vertes R.P., Hoover W.B., Sherman A. (2002). Afferent projections to the medial prefrontal cortex in rats. Soc. Neurosci. Abstr..

[72] Vertes R.P. (2004). Differential projections of the infralimbic and prelimbic cortex in the rat. Synapse.

[73] Laroche S., Jay T.M., Thierry A. (1990). Long-term potentiation in the prefrontal cortex following stimulation of the hippocampal CA1/subicular region. Neurosci. Lett..

[74] Rajasethupathy P., Sankaran S., Marshel J.H., Kim C.K., Ferenczi E., Lee S.Y., Deisseroth K. (2015). Projections from neocortex mediate top-down control of memory retrieval. Nature.

[75] Dolorfo C.L., Amaral D.G. (1998). Entorhinal cortex of the rat: Topographic organization of the cells of origin of the perforant path projection to the dentate gyrus. J. Comp. Neurol..

[76] Ferino F., Thierry A.M., Glowinski J. (1987). Anatomical and electrophysiological evidence for a direct projection from Ammon’s horn to the medial prefrontal cortex in the rat. Exp. Brain Res..

[77] Jay T.M., Thierry A.M., Wiklund L., Glowinski J. (1992). Excitatory Amino Acid Pathway from the Hippocampus to the Prefrontal Cortex. Contribution of AMPA Receptors in Hippocampo-prefrontal Cortex Transmission. Eur. J. Neurosci..

[78] Zhan Y. (2015). Theta frequency prefrontal–hippocampal driving relationship during free exploration in mice. Neuroscience.

[79] Hallock H.L., Wang A., Griffin A.L. (2016). Ventral Midline Thalamus Is Critical for Hippocampal–Prefrontal Synchrony and Spatial Working Memory. J. Neurosci..

[80] O’Neill P.K., Gordon J.A., Sigurdsson T. (2013). Theta oscillations in the medial prefrontal cortex are modulated by spatial working memory and synchronize with the hippocampus through its ventral subregion. J. Neurosci..

[81] De Hoz L., Knox J., Morris R.G. (2003). Longitudinal axis of the hippocampus: Both septal and temporal poles of the hippocampus support water maze spatial learning depending on the training protocol. Hippocampus.

[82] McDonald R.J., Jones J., Richards B., Hong N.S. (2006). A double dissociation of dorsal and ventral hippocampal function on a learning and memory task mediated by the dorso-lateral striatum. Eur. J. Neurosci..

[83] Ferbinteanu J., Ray C., McDonald R.J. (2003). Both dorsal and ventral hippocampus contribute to spatial learning in Long–Evans rats. Neurosci. Lett..

[84] Moser E., Moser M.B., Andersen P. (1993). Spatial learning impairment parallels the magnitude of dorsal hippocampal lesions, but is hardly present following ventral lesions. J. Neurosci..

[85] Moser M.B., Moser E.I. (1998). Functional differentiation in the hippocampus. Hippocampus.

[86] Kesner R.P., Hunsaker M.R., Ziegler W. (2011). The role of the dorsal and ventral hippocampus in olfactory working memory. Neurobiol. Learn. Mem..

[87] Rogers J.L., Hunsaker M.R., Kesner R.P. (2006). Effects of ventral and dorsal CA1 subregional lesions on trace fear conditioning. Neurobiol. Learn. Mem..

[88] Rudy J.W., Matus-Amat P. (2005). The ventral hippocampus supports a memory representation of context and contextual fear conditioning: Implications for a unitary function of the hippocampus. Behav. Neurosci..

[89] Yoon T., Otto T. (2007). Differential contributions of dorsal vs. ventral hippocampus to auditory trace fear conditioning. Neurobiol. Learn. Mem..

[90] Nadel L. (1968). Dorsal and ventral hippocampal lesions and behavior. Physiol. Behav..

[91] Maurer A.P., VanRhoads S.R., Sutherland G.R., Lipa P., McNaughton B.L. (2005). Self-motion and the origin of differential spatial scaling along the septo-temporal axis of the hippocampus. Hippocampus N. Y. Churchill Livingstone.

[92] Kjelstrup K.B., Solstad T., Brun V.H., Hafting T., Leutgeb S., Witter M.P., Moser M.B. (2008). Finite scale of spatial representation in the hippocampus. Science.

[93] Royer S., Sirota A., Patel J., Buzsáki G. (2010). Distinct representations and theta dynamics in dorsal and ventral hippocampus. J. Neurosci..

[94] Keinath A.T., Wang M.E., Wann E.G., Yuan R.K., Dudman J.T., Muzzio I.A. (2014). Precise spatial coding is preserved along the longitudinal hippocampal axis. Hippocampus.

[95] Jones M.W., Wilson M.A. (2005). Phase precession of medial prefrontal cortical activity relative to the hippocampal theta rhythm. Hippocampus.

[96] Hyman J.M., Zilli E.A., Paley A.M., Hasselmo M.E. (2005). Medial prefrontal cortex cells show dynamic modulation with the hippocampal theta rhythm dependent on behavior. Hippocampus.

[97] Kyd R.J., Bilkey D.K. (2003). Prefrontal cortex lesions modify the spatial properties of hippocampal place cells. Cereb. Cortex.

[98] Kyd R.J., Bilkey D.K. (2005). Hippocampal place cells show increased sensitivity to changes in the local environment following prefrontal cortex lesions. Cereb. Cortex.

[99] Hok V., Chah E., Save E., Poucet B. (2013). Prefrontal cortex focally modulates hippocampal place cell firing patterns. J. Neurosci. Off. J. Soc. Neurosci..

[100] Johnson A., Redish A.D. (2007). Neural ensembles in CA3 transiently encode paths forward of the animal at a decision point. J. Neurosci..

[101] Burghardt N.S., Park E.H., Hen R., Fenton A.A. (2012). Adult-born hippocampal neurons promote cognitive flexibility in mice. Hippocampus.

[102] Hok V., Chah E., Reilly R.B., O’Mara S.M. (2012). Hippocampal dynamics predict inter individual cognitive differences in rats. J. Neurosci..

[103] Scoville W.B., Milner B. (1957). Loss of recent memory after bilateral hippocampal lesions. J. Neurol. Neurosurg. Psychiatry.

[104] Yamaguchi Y., Aota Y., Sasto N., Wagatsuma H., Wu Z. (2004). Synchronization of neural oscillations as a possible mechanism underlying episodic memory: A study of theta rhythm in the hippocampus. J. Integr. Neurosci..

[105] Squire L.R., Spanis C.W. (1984). Long gradient of retrograde amnesia in mice: Continuity with the findings in humans. Behav. Neurosci..

[106] Takehara-Nishiuchi K., McNaughton B.L. (2008). Spontaneous changes of neocortical code for associative memory during consolidation. Science.

[107] Takehara K., Kawahara S., Kirino Y. (2003). Time-dependent reorganization of the brain components underlying memory retention in trace eyeblink conditioning. J. Neurosci..

[108] Wiig K.A., Bilkey D.K. (1994). Perirhinal cortex lesions in rats disrupt performance in a spatial DNMS task. Neuroreport.

[109] Rothschild G., Eban E., Frank L.M. (2016). A cortical-hippocampal-cortical loop of information processing during memory consolidation. Nat. Neurosci..

[110] Mishkin M. (1982). A memory system in the monkey. Philos. Trans. R. Soc. Lond. B Biol. Sci..

[111] Tonegawa S., Liu X., Ramirez S., Redondo R. (2015). Memory engram cells have come of age. Neuron.

[112] Wang H.X., Gerkin R.C., Nauen D.W., Bi G.Q. (2005). Coactivation and timing-dependent integration of synaptic potentiation and depression. Nat. Neurosci..

[113] Krug M., Lössner B., Ott T. (1984). Anisomycin blocks the late phase of long-term potentiation in the dentate gyrus of freely moving rats. Brain Res. Bull..

[114] Schafe G.E., Nadel N.V., Sullivan G.M., Harris A., Le Doux J.E. (1999). Memory consolidation for contextual and auditory fear conditioning is dependent on protein synthesis, PKA, and MAP kinase. Learn. Mem..

[115] Apergis-Schoute A.M., Dębiec J., Doyere V., Le Doux J.E., Schafe G.E. (2005). Auditory fear conditioning and long-term potentiation in the lateral amygdala require ERK/MAP kinase signaling in the auditory thalamus: A role for presynaptic plasticity in the fear system. J. Neurosci..

[116] Nabavi S., Fox R., Proulx C.D., Lin J.Y., Tsien R.Y., Malinow R. (2014). Engineering a memory with LTD and LTP. Nature.

[117] Ding H.K., Teixeira C.M., Frankland P.W. (2008). Inactivation of the anterior cingulate cortex blocks expression of remote, but not recent, conditioned taste aversion memory. Learn. Mem..

[118] Maviel T., Durkin T.P., Menzaghi F., Bontempi B. (2004). Sites of neocortical reorganization critical for remote spatial memory. Science.

[119] Meunier M., Destrade C. (1986). Paradoxical transitory facilitation of performance in the Hebb-Williams labyrinth after lesion of the cingulate cortex in mice. C. R. Acad. Sci. III.

[120] Liu F., Zheng X.L., Li B.M. (2009). The anterior cingulate cortex is involved in retrieval of long-term/long-lasting but not short-term memory for step-through inhibitory avoidance in rats. Neurosci. Lett..

[121] Poucet B. (1997). Searching for spatial unit firing in the prelimbic area of the rat medial prefrontal cortex. Behav. Brain Res..

[122] Niki H., Watanabe M. (1979). Prefrontal and cingulate unit activity during timing behavior in the monkey. Brain Res..

[123] Adhikari A., Topiwala M.A., Gordon J.A. (2011). Single units in the medial prefrontal cortex with anxiety-related firing patterns are preferentially influenced by ventral hippocampal activity. Neuron.

[124] Hyman J.M., Hasselmo M.E., Seamans J.K. (2011). What is the functional relevance of prefrontal cortex entrainment to hippocampal theta rhythms?. Front. Neurosci..

[125] Takehara-Nishiuchi K., Nakao K., Kawahara S., Matsuki N., Kirino Y. (2006). Systems consolidation requires postlearning activation of NMDA receptors in the medial prefrontal cortex in trace eyeblink conditioning. J. Neurosci..

[126] Lee A.K., Wilson M.A. (2002). Memory of sequential experience in the hippocampus during slow wave sleep. Neuron.

[127] Euston D.R., Tatsuno M., McNaughton B.L. (2007). Fast-forward playback of recent memory sequences in prefrontal cortex during sleep. Science.

[128] Peyrache A., Khamassi M., Benchenane K., Wiener S.I., Battaglia F.P. (2009). Replay of rule-learning related neural patterns in the prefrontal cortex during sleep. Nat. Neurosci..

[129] Nádasdy Z., Hirase H., Czurkó A., Csicsvari J., Buzsáki G. (1999). Replay and time compression of recurring spike sequences in the hippocampus. J. Neurosci..

[130] Louie K., Wilson M.A. (2001). Temporally structured replay of awake hippocampal ensemble activity during rapid eye movement sleep. Neuron.

[131] Carr M.F., Jadhav S.P., Frank L.M. (2011). Hippocampal replay in the awake state: A potential substrate for memory consolidation and retrieval. Nat. Neurosci..

[132] Benchenane K., Tiesinga P.H., Battaglia F.P. (2011). Oscillations in the prefrontal cortex: A gateway to memory and attention. Curr. Opin. Neurobiol..

[133] Takashima A., Jensen O., Oostenveld R., Maris E., Van de Coevering M., Fernandez G. (2006). Successful declarative memory formation is associated with ongoing activity during encoding in a distributed neocortical network related to working memory: A magnetoencephalography study. Neuroscience.

[134] Mulder A.B., Nordquist R.E., Örgüt O., Pennartz C.M. (2003). Learning-related changes in response patterns of prefrontal neurons during instrumental conditioning. Behav. Brain Res..

[135] Alberini C.M. (2011). The role of reconsolidation and the dynamic process of long-term memory formation and storage. Front. Behav. Neurosci..

[136] Cohen R.A., Kaplan R.F., Moser D.J., Jenkins M.A., Wilkinson H. (1999). Impairments of attention after cingulotomy. Neurology.

[137] Wheeler A.L., Teixeira C.M., Wang A.H., Xiong X., Kovacevic N., Lerch J.P., Frankland P.W. (2013). Identification of a functional connectome for long-term fear memory in mice. PLoS Comput. Biol..

[138] Teixeira C.M., Pomedli S.R., Maei H.R., Kee N., Frankland P.W. (2006). Involvement of the anterior cingulate cortex in the expression of remote spatial memory. J. Neurosci..

[139] Frankland P.W., Bontempi B. (2005). The organization of recent and remote memories. Nat. Rev. Neurosci..

[140] Insel N., Takehara-Nishiuchi K. (2013). The cortical structure of consolidated memory: A hypothesis on the role of the cingulate–entorhinal cortical connection. Neurobiol. Learn. Mem..

[141] Hasselmo M.E. (2005). A model of prefrontal cortical mechanisms for goal-directed behavior. J. Cognit. Neurosci..

[142] Otto T., Eichenbaum H. (1992). Neuronal activity in the hippocampus during delayed non-match to sample performance in rats: Evidence for hippocampal processing in recognition memory. Hippocampus.

[143] Jung M.W., McNaughton B.L. (1993). Spatial selectivity of unit activity in the hippocampal granular layer. Hippocampus.

[144] Ma L., Hyman J.M., Lindsay A.J., Phillips A.G., Seamans J.K. (2014). Differences in the emergent coding properties of cortical and striatal ensembles. Nat. Neurosci..

[145] Warren C.M., Hyman J.M., Seamans J.K., Holroyd C.B. (2015). Feedback-related negativity observed in rodent anterior cingulate cortex. J. Physiol.-Paris.

[146] De Saint Blanquat P., Hok V., Alvernhe A., Save E., Poucet B. (2010). Tagging items in spatial working memory: A unit-recording study in the rat medial prefrontal cortex. Behav. Brain Res..

[147] De Bruin J.P., Sànchez-Santed F., Heinsbroek R.P., Donker A., Postmes P. (1994). A behavioural analysis of rats with damage to the medial prefrontal cortex using the Morris water maze: Evidence for behavioural flexibility, but not for impaired spatial navigation. Brain Res..

[148] Hart E.E., Stolyarova A., Conoscenti M.A., Minor T.R., Izquierdo A. (2016). Rigid patterns of effortful choice behavior after acute stress in rats. Stress.

[149] Lapiz M.D.S., Morilak D.A. (2006). Noradrenergic modulation of cognitive function in rat medial prefrontal cortex as measured by attentional set shifting capability. Neuroscience.

[150] Brady A.M., Floresco S.B. (2015). Operant Procedures for Assessing Behavioral Flexibility in Rats. J. Vis. Exp..

[151] Xu H., Zhang Y., Zhang F., Yuan S., Shao F., Wang W. (2016). Effects of Duloxetine Treatment on Cognitive Flexibility and BDNF Expression in the mPFC of Adult Male Mice Exposed to Social Stress during Adolescence. Front. Mol. Neurosci..

[152] Floresco S.B. (2013). Prefrontal dopamine and behavioral flexibility: Shifting from an “inverted-U” toward a family of functions. Front. Neurosci..

[153] Ma L., Hyman J.M., Phillips A.G., Seamans J.K. (2014). Tracking progress toward a goal in corticostriatal ensembles. J. Neurosci..

[154] Ragozzino M.E., Detrick S., Kesner R.P. (1999). Involvement of the prelimbic–infralimbic areas of the rodent prefrontal cortex in behavioral flexibility for place and response learning. J. Neurosci..

[155] Jo Y.S., Park E.H., Kim I.H., Park S.K., Kim H., Kim H.T., Choi J.S. (2007). The medial prefrontal cortex is involved in spatial memory retrieval under partial-cue conditions. J. Neurosci..

[156] Dias R., Aggleton J.P. (2000). Effects of selective excitotoxic prefrontal lesions on acquisition of nonmatching-and matching-to-place in the T-maze in the rat: Differential involvement of the prelimbic–infralimbic and anterior cingulate cortices in providing behavioural flexibility. Eur. J. Neurosci..

[157] Granon S., Poucet B. (1995). Medial prefrontal lesions in the rat and spatial navigation: Evidence for impaired planning. Behav. Neurosci..

[158] Fenton A.A., Lytton W.W., Barry J.M., Lenck-Santini P.P., Zinyuk L.E., Kubík Š., Olypher A.V. (2010). Attention-like modulation of hippocampus place cell discharge. J. Neurosci..

[159] Loftus E.F., Loftus G.R. (1980). On the permanence of stored information in the human brain. Am. Psychol..

[160] Weible A.P. (2013). Remembering to attend: The anterior cingulate cortex and remote memory. Behav. Brain Res..

[161] Place R., Farovik A., Brockmann M., Eichenbaum H. (2016). Bidirectional prefrontal-hippocampal interactions support context-guided memory. Nat. Neurosci..

[162] Weible A.P., Rowland D.C., Monaghan C.K., Wolfgang N.T., Kentros C.G. (2012). Neural correlates of long-term object memory in the mouse anterior cingulate cortex. J. Neurosci..

